# A recurrent *de novo* MAX p.Arg60Gln variant causes a syndromic overgrowth disorder through differential expression of c-Myc target genes

**DOI:** 10.1016/j.ajhg.2023.11.010

**Published:** 2023-12-22

**Authors:** Erica L. Harris, Vincent Roy, Martin Montagne, Ailsa M.S. Rose, Helen Livesey, Margot R.F. Reijnders, Emma Hobson, Francis H. Sansbury, Marjolein H. Willemsen, Rolph Pfundt, Daniel Warren, Vernon Long, Ian M. Carr, Han G. Brunner, Eamonn G. Sheridan, Helen V. Firth, Pierre Lavigne, James A. Poulter

**Affiliations:** 1 Division of Molecular Medicine, Leeds Institute of Medical Research, University of Leeds, Leeds, UK; 2 Département de Biochimie et Génomique Fonctionnelle, PROTÉO et Institut de Pharmacologie de Sherbrooke. University of Sherbrooke, Sherbrooke, QC, Canada; 3 Leeds Teaching Hospitals NHS Trust, Leeds, UK; 4 Department of Clinical Genetics, Erasmus Medical Center, Rotterdam, the Netherlands; 5 All Wales Medical Genomics Service, NHS Wales Cardiff and Vale University Health Board, Cardiff, UK; 6 Department of Human Genetics, Radboud University Medical Center, Nijmegen, the Netherlands; 7 Addenbrooke’s Hospital, Cambridge University Hospitals, Cambridge, UK; 8 Wellcome Sanger Institute, Hinxton, Cambridge, UK

**Keywords:** MAX, MYC, CCND2, b-HLH-LZ, macrocephaly, polydactyly

## Abstract

Cyclin D2 (CCND2) stabilization underpins a range of macrocephaly-associated disorders through mutation of CCND2 or activating mutations in upstream genes encoding PI3K-AKT pathway components. Here, we describe three individuals with overlapping macrocephaly-associated phenotypes who carry the same recurrent *de novo* c.179G>A (p.Arg60Gln) variant in Myc-associated factor X (MAX). The mutation, located in the b-HLH-LZ domain, causes increased intracellular CCND2 through increased transcription but it does not cause stabilization of CCND2. We show that the purified b-HLH-LZ domain of MAX^Arg60Gln^ (Max^∗Arg60Gln^) binds its target E-box sequence with a lower apparent affinity. This leads to a more efficient heterodimerization with c-Myc resulting in an increase in transcriptional activity of c-Myc in individuals carrying this mutation. The recent development of Omomyc-CPP, a cell-penetrating b-HLH-LZ-domain c-Myc inhibitor, provides a possible therapeutic option for MAX^Arg60Gln^ individuals, and others carrying similar germline mutations resulting in dysregulated transcriptional c-Myc activity.

## Teaser

Harris et al. describe three individuals with overlapping macrocephaly-associated phenotypes carrying a recurrent *de novo* c.179G>A (p.Arg60Gln) variant in Myc-associated factor X (MAX). MAX^Arg60Gln^ binds its target E-box sequence with a lower affinity, meaning more efficient heterodimerization with c-Myc and dysregulated transcriptional activity. c-Myc inhibitors provide a possible therapeutic option for individuals carrying this variant.

## Introduction

The *Myc-associated factor X* (*MAX*) gene (MIM: 154950) encodes a basic-region-helix-loop-helix-leucine zipper (b-HLH-LZ) transcription factor that promotes DNA binding and transcriptional activation as a Myc/Max heterodimer.^1^^,^^2^ MAX can also form a homodimeric b-HLH-LZ and bind DNA with similar affinities and specificity. The canonical enhancer (E)-box (CACGTG) is their preferred target and is found at the promoters of many c-Myc target genes, which include those encoding cyclins and cyclin-dependent kinases (CDKs).^3^ Over expression of c-Myc is observed in many cancers and leads to amplification of transcriptional profiles associated with cellular proliferation.^4^ In contrast, the MAX homodimer acts as an antagonist by binding to canonical E-box sequences and inhibiting the transcription of c-Myc target genes.^5^

While somatic inactivating mutations in *MAX* have been identified in a number of cancers,^6^^,^^7^ to date few germline pathogenic variants in *MAX* have been identified. Germline loss-of-function mutations in *MAX* have been shown to predispose individuals for hereditary phaeochromocytoma (PCC); however, these only resulted in disease if loss of heterozygosity of the wild-type allele also occurs due to uniparental disomy, resulting in complete absence of MAX in tumors.^8^^,^^9^

Here, we describe three individuals who each share a recurrent *de novo* germline variant in *MAX*, resulting in a p.Arg60Gln substitution (p.Arg60Gln) in the loop of the b-HLH-LZ domain. Affected individuals have a complex disorder consisting primarily of macrocephaly, polydactyly, and delayed ophthalmic development. In the presence of the p.Arg60Gln mutation, we found differential expression of c-Myc target genes, including cyclin D2 (*CCND2*) (MIM: 123833), a gene previously associated with a macrocephaly and polydactyly phenotype (MIM: 615938). Electrophoretic mobility shift assay (EMSA) and circular dichroism (CD)-spectral analysis revealed the purified b-HLH-LZ domain of MAX^Arg60Gln^ (Max^∗Arg60Gln^) dimerized more readily as a heterodimeric and specific DNA complex with c-Myc than Max^∗WT^. Furthermore, Max^∗Arg60Gln^ bound its target E-box sequence with a lower apparent affinity than Max^∗WT^ resulting in aberrant transcriptional activity of c-Myc in individuals carrying this mutation. The recent development of Omomyc-CPP, a cell-penetrating b-HLH-LZ domain that inhibits c-Myc-associated transcription, provides a possible therapeutic option for individuals with the MAX^Arg60Gln^ variant, as well as others carrying similar germline mutations resulting in altered transcriptional c-Myc activity.

## Subjects and methods

### Clinical ascertainment and genetic analysis

Individuals 1 and 2 (P1 and P2) were both sequenced as part of the Deciphering Developmental Disorders (DDD) study.^10^^,^^11^ Individual 3 (P3) was sequenced as part of a large cohort in Nijmegen, the Netherlands.^12^ Individuals and their family members were recruited following informed consent and under ethical approval from the Yorkshire & The Humber - Leeds East Research Ethics Committee (REC ref. no. 18/YH/0070) in accordance with the principles outlined in the declaration of Helsinki. DNA samples were obtained via venous blood samples using conventional techniques.

### Cell culture and MAX vector generation

HEK293 cells (ECACC) were cultured in Dulbecco’s modified Eagle’s medium (DMEM), supplemented with 10% fetal calf serum, at 37°C in 5% CO_2_. The c.179G>A (p.Arg60Gln) variant was introduced into the wild-type (WT) *MAX* sequence in pDONR223 (Addgene #82888) via the Q5 site-directed mutagenesis kit (NEB), according to the manufacturer’s instructions. Both wild-type and mutant plasmids were cloned into the pDEST-510 destination vector via the Gateway cloning system (ThermoFisher Scientific), and the sequence was verified by Sanger sequencing with the BigDye Terminator kit v3.1.

### Western blotting

10 μg of protein from whole-cell lysates were separated by SDS-PAGE and transferred onto a polyvinylidene difluoride (PVDF) membrane via the Bolt western-blotting system (ThermoFisher Scientific). The membrane was subsequently blocked in 5% skimmed milk overnight and incubated in a primary antibody in 1% skimmed milk, washed three times in 1× PBS, incubated in a secondary antibody in 0.1% skimmed milk for 1 h, and then washed three times in 1× PBS. Bound antibodies were viewed by the use of SuperSignal West Femto Chemiluminescent substrate on a Bio-Rad Chemi-Doc imaging system with a chemiluminescent filter. After imaging, blots were stripped of antibody with Restore Western Blot Stripping Buffer (ThermoFisher Scientific). Primary antibodies were all from Cell Signaling Technology and used at a dilution of 1/1,000 unless otherwise noted: rabbit-cyclinD2 (#3741), rabbit-CDK4 (#12790), rabbit-MAX (#4739), mouse-beta-actin (1/10,000, Ambion), rabbit-phospho-retinoblastoma (RB) (Ser807/811) XP (#8516), and mouse-RB (#9309). Secondary antibodies used were goat anti-rabbit HRP (#7074) or horse anti-mouse HRP (#7076) used at a final dilution of 1/5,000.

### Cycloheximide assay

HEK293 cells were seeded at 5 × 10^5^ cells per well of a 6-well plate. After 24 h, cells were transfected using a 1:3 ratio of PEI (Merck): plasmid in Opti-Mem reduced serum media. A negative control without any plasmid DNA was also performed for each experiment. 24 h after transfection, Cycloheximide (Merck) was added to a final concentration of 10 μg/mL to the required wells and incubated for 1.5 h. Cell lysis was performed using NP-40 lysis buffer (ThermoFisher Scientific) containing 1× Halt Protease and Phosphatase Inhibitor Cocktail (EDTA free) and protein quantified using the DC Protein Assay kit (Bio-rad). Western blotting was performed as above.

### Real-time quantitative PCR

Total RNA was extracted from HEK293 cells by using the Monarch Total RNA Miniprep Kit (New England Biolabs), and first-strand cDNA synthesis was performed with the ProtoScript First Strand cDNA Synthesis kit (New England Biolabs). Real-time quantitative PCR (qPCR) was performed with the TaqMAN assay system (ThermoFisher Scientific), comprising 2× TaqMAN fast advanced mastermix and FAM-labeled probes against *CCND2* (Hs00153380_m1) and *GAPDH* (Hs99999905_m1). Samples were run on a QuantStudio 5 Real-Time PCR System, and the resulting data analyzed by using ThermoFisher Connect software. Relative gene expression was calculated by using the ΔΔC_T_ method, relative to *GAPDH,* from which log fold change was calculated with log_2_^−ΔΔCT^. Data are expressed as the means ± SEM of three biological replicates.

### Molecular modeling

All modeling and molecular rendering was done with the open-source version 1.7.6.0 of Pymol.^13^ The crystal structure of the apo-form of the heterodimeric b-HLH-LZ (6G6J)^14^ was used as a template for the generation of the model of the c-Myc^∗^:Max^∗Arg60Gln^ heterodimeric HLH. The same side-chain conformation of Gln60 was obtained from the Pymol rotamer library and selected to optimize the packing without introducing tertiary and quaternary steric clashes. The conformation of the K^392^ side chain was manually obtained by setting χ1, χ2, χ3, and χ4 to *gauche +*, *trans*, *gauche –*, and *trans*, respectively, in order to optimize the packing without introducing steric clashes.

### Protein expression and purification

The c-Myc b-HLH-LZ (c-Myc^∗^) was expressed and purified as previously described.^15^ Max^∗WT^ or Max^∗Arg60Gln^ were expressed and purified as described in Maltais et al.^16^ After lyophilization, the b-HLH-LZs were kept lyophilized at −20°C and solubilized in Myc buffer (50 mM NaCl, 50 mM NaH_2_PO_4_ [pH 5.5]) for c-Myc^∗^ or PBS for Max^∗^ at a final concentration of 1 mM before use.

### EMSA

The strands of the specific probe, 5′-ATT ACC CAC GTG TCC T^∗^AC-3′ and 5′-GTA GGA CAC GTG GGT^∗^ AAT-3′ (with the E-box sequence underlined and the asterisk indicating a nucleobase bearing a fluorescein isothiocyanate label for fluorescently labeled probes), and the non-specific probe, 5′-ATT ACC TCC GGA TCC T^∗^AC-3′ and 5′-GTA GGA TCC GGA GGT^∗^ AAT-3′ (Integrated DNA Technologies), were solubilized in 10 mM Tris (pH 8.0) to a concentration of 2 mM. DNA duplexes were diluted to 6.25 μM in 10 mM Tris pH 8.0 and then to 1.25 μM in ddH_2_O. Max^∗^ (WT and Arg60Gln) was diluted to 50 μM in PBS and then further diluted to 5 μM in ddH_2_O in the presence of TCEP at 200 μM and incubated overnight at room temperature. c-Myc^∗^ was diluted in Myc buffer to a concentration of 375 μM and then further diluted to a concentration of 2.5 μM in 1× EMSA binding buffer (20 mM Tris [pH 8.0], 75 mM KCl, 2.5 mM tris(2-carboxyethyl)phosphine (TCEP), 25 μg/mL BSA, and 5% glycerol). C-Myc^∗^ and Max^∗^ constructs were then incubated for 15 min at 37°C, mixed together at a final concentration of 250 and 500 nM, and further incubated for 15 min at 37°C. The fluorescently labeled E-box probe was added to a final concentration of 250 nM, and unlabeled non-specific DNA was added to various concentrations, ranging from 0 to 2,000 nM. All sample volumes were made to 10 μL such that the buffer composition was constant in each sample and then further incubated for 30 min at 37°C before being separated by electrophoresis on 6% native PAGE in 1x TA buffer pH 8.0 at 100 V for 40 min. The resulting images were obtained with a Molecular Imager VersaDoc MP4000 system.

### Circular dichroism

All circular dichroism (CD) measurements were performed on a Jasco J-810 spectropolarimeter equipped with a Peltier-type thermostat. The instrument was routinely calibrated using an aqueous solution of *d*-10-(+)-camphorsulfonic acid at 290.5 nm. Samples were prepared as follows: Max^∗^ (either WT or Arg60Gln) was diluted in 100 μL 2X CD buffer (40 mM KCl, 11.4 mM K_2_HPO_4_, and 28.6 mM KH_2_PO_4_ [pH 6.8]) and the volume adjusted to 106 μL with PBS. 10 μL 16 mM TCEP was added and the volume was further adjusted to 192 μL with ddH_2_O before samples were incubated overnight at room temperature. After reduction, Myc was added and the volume adjusted to 198 μL with Myc buffer. After a 10 min incubation at room temperature, 1 or 2 μL of 2 mM specific or non-specific DNA duplexes (same unlabeled DNA duplexes used in EMSA) in 10 mM Tris pH 8.0 were added and the volume adjusted to 200 μL with 10 mM Tris (pH 8.0). Samples were further incubated for 10 min at room temperature and transferred to a 1-mm path length quartz cuvette. Spectra were recorded from 250 to 195 nm at 0.1 nm intervals through the accumulation of 10 spectra at 25°C. Thermal denaturations were recorded at 222 nm from 5°C to 95°C at a heating rate of 1°C/min.

### mRNA sequencing

Transcriptome libraries were created from 100 ng of mRNA using the Illumina TruSeq library kit according to the manufacturer’s protocols. Pooled libraries were sequenced on a NextSeq 500 using a 15 bp paired-end protocol. The resulting fastq files were trimmed using cutadapt (v1.16) and quality checked using FastQC. All reads passing QC were subsequently aligned to the GRCh38 build of the human genome with STAR (v2.7.10b) and read counts per exon obtained by using Rsubread. Differential gene expression (DGE) was performed with DESeq2.

### Statistical analyses

Statistical analyses were performed with Prism 9 software. Three independent biological replicates were used for statistical calculations unless otherwise stated. All statistical tests performed were unpaired two sided, and a p value of <0.05 was considered statistically significant. Error bars represent standard deviation.

## Results

### Identification of the recurrent MAX p.Arg60Gln *de novo* variant in two independent datasets

**Figure 1:**
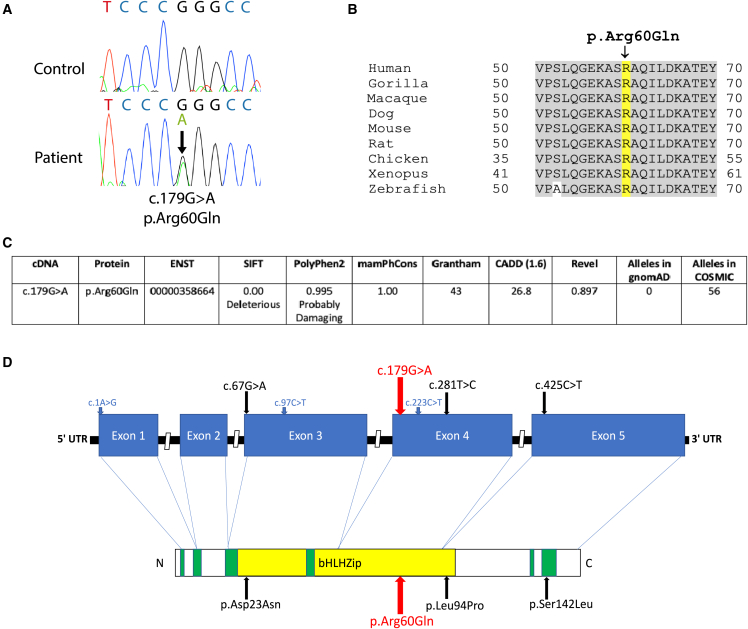
The recurrent *de novo* c.179G>A (p.Arg60Gln) variant in MAX causes a macrocephaly- and polydactyly-associated syndrome (A) Representative electropherogram showing c.179G>A pathogenic variant in individual P1 compared to an unrelated control. The c.179G>A variant is highlighted by the arrow. (B) Conservation analysis of the p.Arg60Gln variant in MAX shows the Arginine-60 residue, and the surrounding amino acid sequence is conserved in all organisms tested. (C) Variant information and bioinformatic predictions for the c.179G>A (p.Arg60Gln) variant. (D) 2D genomic and proteomic structure of MAX showing the location of the c.179G>A (p.Arg60Gln) variant. Additional published truncating and missense variants predisposing to hereditary pheochromocytoma are indicated in blue and black arrows, respectively.^8^^,^^9^

We observed in the DDD dataset^11^ two individuals (P1, DDD: 304967 and P2, DDD: 303133) with identical *de novo* mutations (DNMs) in the tumor suppressor gene *MAX*, resulting in an arginine (Arg)-to-glutamine (Gln) substitution at residue 60 (c.179G>A [GenBank: NM_002382.5] [p.Arg60Gln]) and overlapping phenotypes of polydactyly and macrocephaly, a rare combination in DDD observed in 18/13,610 (0.132%) participants. Interrogation of a second dataset^12^ identified a third individual (P3) with the same *de novo* p.Arg60Gln variant with a phenotype that also included polydactyly. The variant was confirmed by Sanger sequencing in all available affected individuals and family members (Figure 1A), and conservation analysis in DECIPHER showed the Arg60 residue, and the flanking residues, to be fully conserved to zebrafish (Figure 1B). The p.Arg60Gln variant was not present in approximately 251,356 alleles in gnomAD nor is it present in dbSNP (v.153). Interrogation of the catalog of somatic mutations in cancer (COSMIC), however, revealed the variant has been identified 56 times in tumor tissue (COSM166665, COSV52414939) from five different tissues, most commonly kidney (17) followed by central nervous system (10), large intestine (9), endometrium (7), and hematological/lymphoid (3). Analysis of the variant predicted it to be pathogenic in all software tested, including CADD, REVEL, SIFT, PolyPhen2, and Grantham (Figure 1C). We therefore sought to confirm if the recurrent p.Arg60Gln variant was the cause of disease in the three individuals identified.

### The MAX p.Arg60Gln alteration causes a syndromic overgrowth disorder

**Figure 2:**
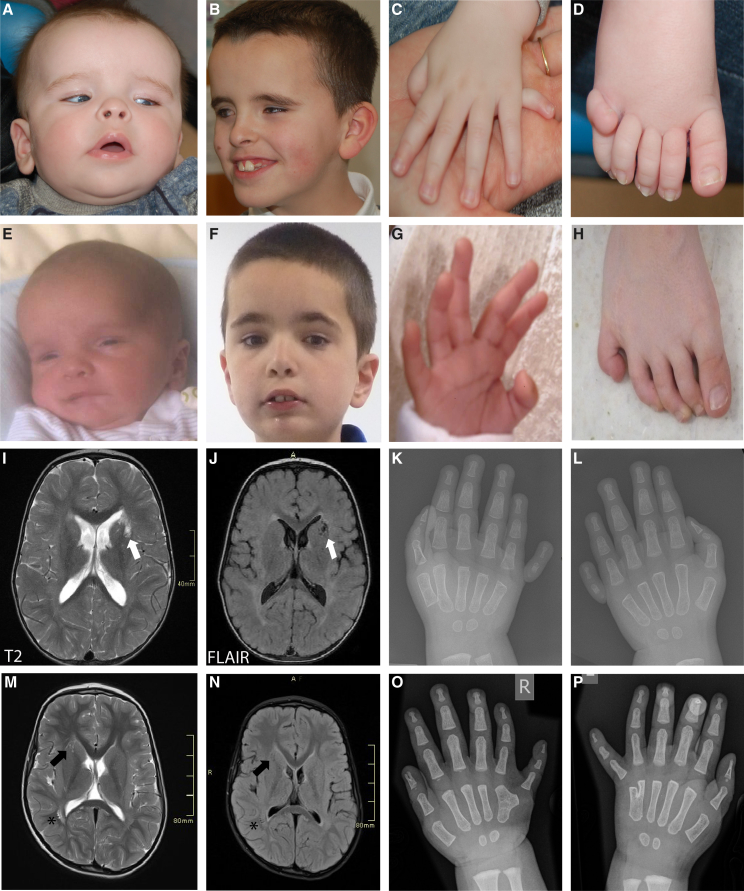
Clinical phenotype of individuals with the *de novo* Arg60Gln MAX variant Photographs and brain MRI scans of P1 (A–D) and P2 (E–H) are shown. Photographs show macrocephaly with a prominent forehead at <1 year of age (A and E), aged 7 (B) and 10 years (F), and postaxial polydactyl of the hands (C and G) and of the feet (D and H). Axial MRI T2 and FLAIR images demonstrate prominent perivascular spaces in the basal ganglia on the left in P1 (I and J: white arrow) and on the right in P2 (M and N: black arrow). P2 also has prominent perivascular spaces more posteriorly adjacent to the trigone of the right lateral ventricle (^∗^). Hand X-rays of P1 (K–L) and P2 (O–P) show post-axial polydactyly of both the right (K and O) and left (L and P) hands. The parents of all subjects gave informed consent for publication of photographs.

Based on the promising genetic analysis, a more detailed clinical investigation of the individuals was performed. A clinical summary can be found in Table 1. In brief, all three cases had 3- or 4-limb polydactyly, and two of the individuals also had a progressive macrocephaly (P1 and P2, Figure 2). P1 also has bilateral microphthalmia, which is more severe in his left eye and large chorioretinal colobomas, affecting the optic nerve and central vision (Figure S1). He subsequently developed retinal exudation requiring cryotherapy and developed a cataract in his right eye. P1 displayed autistic traits, but his intellectual development was appropriate for his level of visual impairment. P2 is also autistic and has developmental delay. In addition, he displayed delayed visual maturation, with visual inattention diagnosed at 3 months of age, but had normal vision by 44 months of age. A comparison of brain MRI scans between P1 and P2 showed marked similarities, including prominent perivascular spaces in the basal ganglia/peri-caudate region (Figures 2I and 2M). P2 also has prominent perivascular spaces more posteriorly adjacent to the trigone of the right lateral ventricle (Figures 2M and 2N). P3 was found to have a normal occipital frontal circumference (OFC) at birth, and there was no evidence of polymicrogyria on postmortem MRI but showed a swollen brain with decreased gray-white matter differentiation and decreased demarcation of the cortex. Systemically, P3 had 4-limb polydactyly and multiple additional complications, including an atrial septum defect, hypospadias, single umbilical artery, and died 1 h after birth due to bilateral renal agenesis. Neither P1 nor P2 have any known cardiac or renal phenotypes; however, P1 did have a persistent patent foramen ovale, which is now closed.

**Table 1:** Clinical summary of 3 individuals with the MAX c.179G>A (p.Arg60Gln) variant

**Case**	**Sex**	**OFC**^a^**(age, year.month)**	**VMEG**	**Polydactyly**^b^	**ID**	**Ophthalmic**	**Cardiac**	**Additional phenotypes**
P1	M	+1.94 (0.0) +3.4 (7.1)	yes	++/++	appropriate for the level of visual impairment; autistic traits	chorio-retinal colobomas affecting optic nerves and vision; developed exudative retinopathy in both eyes	persistent patent foramen ovale (now closed)	gastro-esophageal reflux, 4 phalanges on left thumb, pectus carinatum
P2	M	+3.2 (2.6) +3.02 (9.9)	no	++/+-	autistic with GDD^c^	delayed visual maturation	no cardiac phenotype	perianal abscesses (developed aged 4 years)
P3	M	Normal (0.0)	no	++/++	N/A	N/A	atrial septal defect	hypospadias, renal agenesis, single umbilical artery, flattened thoracic vertebrae

VMEG, ventriculomegaly; GDD, global developmental delay.

a OFC presented as standard deviations relative to the UK_1990 mean for age and sex.

b Postaxial polydactyly indicated for both hands and both feet (hands/feet), i.e., ++/++ indicates polydactyly on both hands and feet, ++/− indicates on hands only.

c Sat aged 12 months; walked at approx. 24 months; spoke single words aged 5 years; started to speak 3-word sentences aged 5 years 11 months.

### CCND2 is upregulated in the presence of MAX^Arg60Gln^

**Figure 3:**
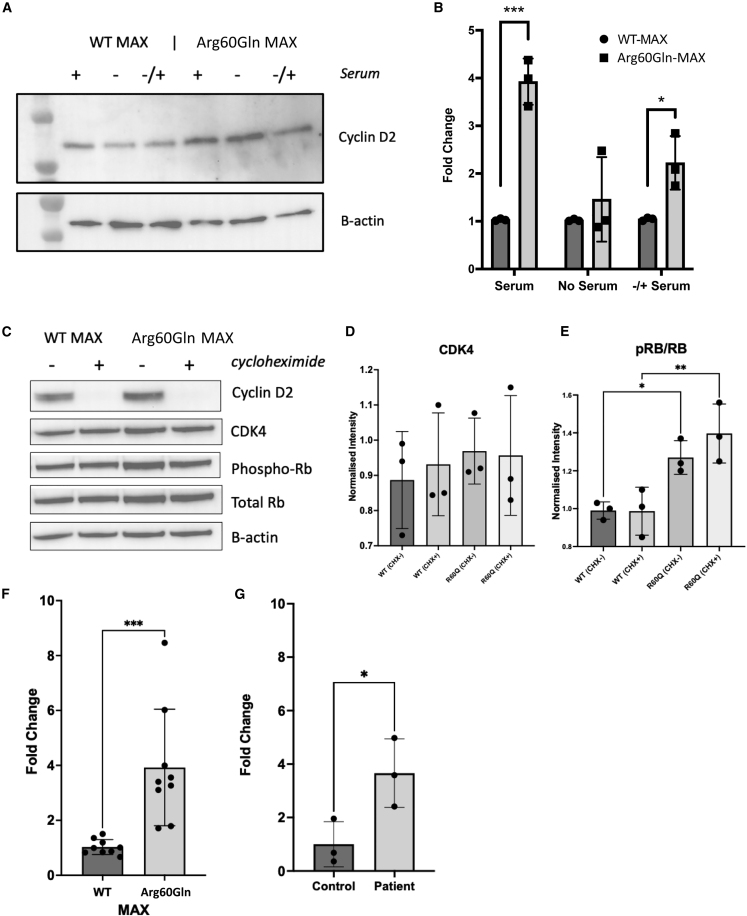
The Arg60Gln variant in MAX results in increased expression of *CCND2* and not CCND2 stabilization (A) Representative Western blot of CCND2 in HEK293 cells transfected with MAX^WT^ or MAX^Arg60Gln^ in the presence (+) or absence (−) of serum or after serum starvation for 24 h and reintroduced for 20 min (−/+). (B) Summary of CCND2 intensities normalized against B-actin. Plots show summary of 3 biological replicates. (C) Representative Western blot of CCND2, CDK4, phosphorylated-RB, and total RB in MAX^WT^ or MAX^Arg60Gln^ transfected cells in the presence or absence of cycloheximide. (D) Summary of CDK4 band intensities normalized against B-actin (n = 3). (E)– Summary of phosphorylated-RB bands relative to total RB (n = 3). (F) Real-time qPCR of MAX^WT^ or MAX^Arg60Gln^ transfected HEK293 cells showing fold change of *CCND2* mRNA normalized against *GAPDH* (n = 3). (G) Real-time qPCR of control and individual P1 *CCND2* mRNA in peripheral blood mononuclear cell RNA normalized against *GAPDH* (n = 3). ^∗^p < 0.05, ^∗∗^p < 0.01, ^∗∗∗^p < 0.001.

Because of the co-occurrence of macrocephaly and polydactyly, a combination of phenotypes often observed in mTOR hyperactivation disorders, which result in CCND2 stabilization,^17^ we hypothesized that the MAX^Arg60Gln^ variant may also result in CCND2 stabilization. We therefore introduced the c.179G>A (p.Arg60Gln) mutation into wild-type MAX (*MAX*^WT^) C-terminally tagged with FLAG. As expected, transfection of *MAX* ^Arg60Gln^, compared to *MAX*^WT^, into HEK293 cells resulted in a significant increase in CCND2 protein (Figure 3A). Furthermore, we found that while serum starving cells for 24 h resulted in a reduction in CCND2 in MAX^WT^ transfected cells, a similar reduction in CCND2 was not observed in MAX^Arg60Gln^ transfected cells (Figure 3B). Analysis of protein lysate from cells treated with cycloheximide, however, revealed CCND2 was not being stabilized in the presence of either MAX^WT^ or MAX^Arg60Gln^, with no significant difference in CCND2 degradation observed (Figure 3C). No difference in levels of CDK4 were observed; however, we saw a significant increase in phosphorylated RB1 (Figures 3C–3E). Instead, real-time qPCR on RNA extracted from transfected cells revealed that these cells had significantly more *CCND2* mRNA than did cells transfected with MAX^WT^ (Figure 3F). This significant increase in *CCND2* mRNA was subsequently confirmed by real-time qPCR of blood RNA from P1 (Figure 3G). Altogether our data indicate that CCND2 is not stabilized but *CCND2* transcription is significantly increased in cells containing MAX^Arg60Gln^, resulting in a significant increase in CCND2 protein and activation of downstream pathways.

### The p.Arg60Gln alteration in MAX leads to a more specific heterodimerization between its b-HLH-LZ and the b-HLH-LZ of c-Myc

**Figure 4:**
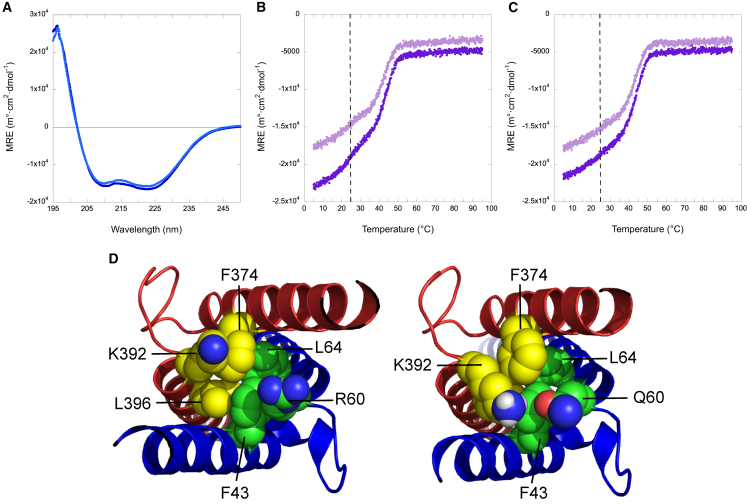
The Arg60Gln alteration leads to a more specific heterodimerization between the b-HLH-LZ of MAX and the b-HLH-LZ of c-Myc (A) Far-UV CD spectra of Max^∗WT^ (dark blue) and Max^∗Arg60Gln^ (light blue) recorded at 20°C, pH 6.8, and 15 μM. The data are reported in mean residue ellipticity (MRE). (B) Temperature denaturation of Max^∗WT^ and c-Myc^∗^ at 10 μM:10 μM (light) and 15 μM:10 μM (dark), respectively. The vertical dashed line represents the apparent T° of the Max^∗^ homodimers. (C) Temperature denaturations of Max^∗Arg60Gln^ and c-Myc^∗^ at 10 μM:10 μM (light) and 15 μM:10 μM (dark), respectively. (D) Cartoon representations of the apo-form of WT heterodimeric HLH of c-Myc^∗^:Max^∗^ (6G6J) displaying the side-chains of Phe43, Arg60, and Leu64 on Max and those of Phe374, Lys392, and Leu396 on c-Myc. Cartoon representation of a model of the Max^∗Arg60Gln^:c-Myc^∗^ heterodimeric HLH. Yellow and green spheres are carbon atoms while red and blue spheres represent oxygen and nitrogen atoms, respectively.

To gain further insights into why MAX^Arg60Gln^ resulted in increased *CCND2* expression, we evaluated the impact of the mutation on the overall structure and homodimerization using an isolated b-HLH-LZ domain of MAX (Max^∗^). The far-UV CD spectra of the WT (Max^∗WT^) and Arg60Gln (Max^∗Arg60Gln^) b-HLH-LZ recorded at 25°C showed typical spectroscopic signatures of a protein composed of a mixture of random and α-helical structures with the two minima centered around 208 and 222 nm (Figure 4A). Given that both Max^∗WT^ and Max^∗Arg60Gln^ display the same helical content indicated that the secondary, tertiary, and quaternary structure of both proteins are similar.

We next evaluated the relative ability of Max^∗WT^ and Max^∗Arg60Gln^ to heterodimerize with c-Myc^∗^. We show the temperature denaturations of Max^∗WT^ and Max^∗Arg60Gln^ in the presence of c-Myc^∗^ to be at molar ratios of 10 μM:10 μM (open circle) and 15 μM:10 μM (filled circle), respectively (Figures 4B and 4C). Two apparent transitions were observed. As described previously,^18^^,^^19^ the first transition corresponds to the denaturation of Max^∗WT^ homodimer (T° = 25°C) and the second to the denaturation of the WT heterodimer (T° = 41°C). Interestingly, for Max^∗Arg60Gln^:c-Myc^∗^, the homodimer transition was not as apparent with only one clear transition (T° = 41°C) corresponding to the Max^∗Arg60Gln^:c-Myc^∗^ heterodimer. The lack of the homodimer transition and population indicates the Max^∗Arg60Gln^ mutant heterodimerizes more efficiently with c-Myc^∗^ than Max^∗WT^. In support of this, molecular modeling revealed that the Oε1 of Gln^60^ can reach within 4 Å of the Hζ of Lys^392^ on c-Myc^∗^ to establish charged H bonds but also reduces electrostatic repulsions with Lys^392^ in the WT heterodimer (Figure 4D).

### The Arg60Gln alteration in Max weakens its affinity for the canonical E-box and leads to a more specific heterodimeric b-HLH-LZ:E-box complex

**Figure 5:**
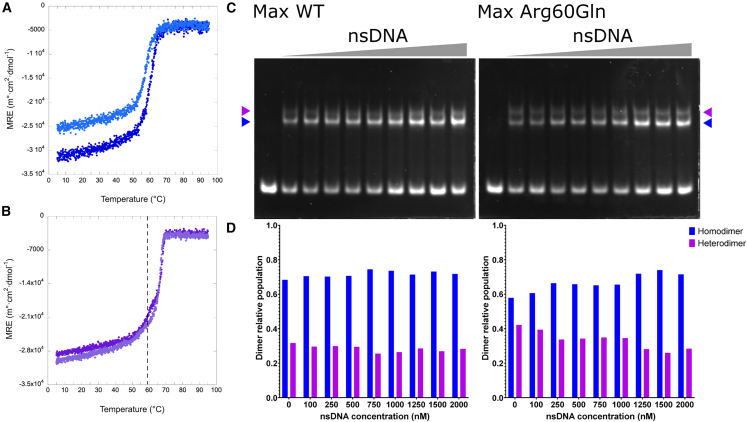
The Arg60Gln mutation weakens the affinity of the b-HLH-LZ of MAX for the canonical E-box but leads to a more specific heterodimeric b-HLH-LZ:E-box complex (A) Temperature denaturation of Max^∗WT^ (dark) and Max^∗Arg60Gln^ (light) in the presence of the canonical E-box at a 10 μM:10 μM molar ratio of protein:DNA duplex, respectively. (B) Temperature denaturations of Max^∗^ WT (dark) and Max^∗^ Arg60Gln (light) in the presence of c-Myc^∗^ and the canonical E-box at molar ratios of Max^∗^:c-Myc:DNA duplex of 15 μM:10 μM:20 μM. The vertical dashed line represents the apparent T° of the Max^∗WT^:E-box complex. (C) Gels of the EMSA of the fluorescently labeled canonical E-box duplex (250 nM) by Max^∗^WT (500 nM):c-Myc^∗^ (250 nM) and Max^∗^ Arg60Gln (500 nM):c-Myc^∗^ (250 nM) in the presence of increasing amount of non-specific duplex DNA (nsDNA) (same length and composition but with the E-box scrambled), respectively. (D) Corresponding bar graphs reporting the relative Max^∗^ homo- (blue) and heterodimeric (purple) populations.

Previous studies have shown the replacement of Arg^60^ by an alanine significantly reduces the binding affinity of the homodimeric b-HLH-LZ to the E-box sequence.^20^ In the structures of the homodimer^21^^,^^22^ and heterodimer^23^ bound to the E-box, the side chain of Arg^60^ contributes to hydrophobic tertiary interactions and forms a salt bridge with a phosphate group through its charged guanidino moiety. Compared to arginine, the glutamine side chain is slightly smaller. However, it has a similar polar surface area (albeit neutral) and a slightly smaller hydrophobic surface area with one less methylene. From the simple replacement of Arg^60^ by a glutamine in the structures of the Max homodimer bound to an E-box (not shown), we noted that while the NH_2_ of the carboxamide can form an H bond with a PO_4_ group, it will lack the electrostatic counterpart. Hence, we hypothesized that this lack of electrostatic interaction will lead to a reduction in affinity for the E-box sequence. As anticipated, we found Max^∗Arg60Gln^ binds the E-box sequence with a lower apparent affinity than Max^∗WT^ (Figure 5A). Furthermore, the Max^∗Arg60Gln^ homodimer is much less stabilized by the presence of the E-box. This is despite the mean residue ellipticity (MRE) of the Max^∗Arg60Gln^ mutant before the transition being much less than that of the Max^∗WT^. Altogether, this suggests that the population of the homodimer bound and with a fully folded basic region is less for the Max^∗Arg60Gln^ compared to the Max^∗WT^ homodimer. Congruently, the transition, which corresponds to the dissociation of the dimer from DNA and into its monomeric components, occurs at a lower temperature (56°C vs. 59°C). This indicates that at the same concentration, the homodimeric Max^∗Arg60Gln^ will be less frequently bound to the E-box than the wild type. We therefore anticipate that Max^∗Arg60Gln^ should be more prone to heterodimerize with c-Myc^∗^ and bind to the E-box sequences as a heterodimer than the WT counterpart.

**Figure 6:**
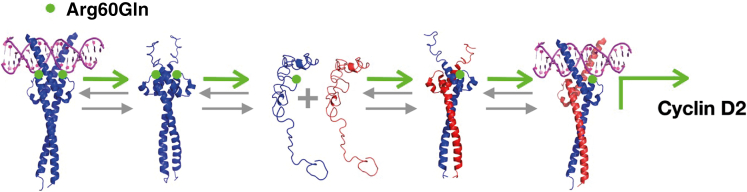
Schematic representation of the effect of the Arg60Gln mutation on Max Max has an increased ability to partition as a heterodimer bound to DNA and activate the transcription of c-Myc target genes.

In order to validate the latter assertion, we ran thermal denaturation monitored by CD of the mixes (15 mM:10 mM) between both constructs of Max^∗^ and c-Myc^∗^ described above but in the presence of the E-box sequence. When exposed to specific DNA, the temperature denaturation of the Max^∗WT^:c-Myc^∗^ (dark) mix displays two distinct transitions corresponding to the population homodimer and heterodimer bound to the E-box sequence, respectively (Figure 5B). However, for Max^∗Arg60Gln^:c-Myc^∗^ (light), the homodimeric complex population is barely apparent, indicating a larger population of heterodimeric complex. This is further supported by the higher helical content as judged by the more negative MRE. In fact, the larger population of homodimer complex in the case of Max^∗WT^ leads to a larger population of free c-Myc^∗^ and hence lower global helical content since the latter does not readily fold into a helical homodimer. In order to further validate the ability of Max^∗Arg60Gln^ to preferentially partition as a heterodimer, we used EMSA. The electrophoretic mobility shifts of the fluorescently labeled E-box (250 nM) by a mixture of Max^∗WT^ and c-Myc^∗^ (1,000 nM:500 nM) and Max^∗Arg60Gln^ and c-Myc^∗^ (1,000 nM:500 nM), respectively, and in the presence of an increasing amount of a non-specific (scrambled E-box from 0 nM to 2,000 nM) are shown in Figure 5C. These ratios were chosen in order to visualize and quantify both the Max^∗^ homodimers and the respective heterodimers as a function of the concentration of competing and non-specific DNA. The heterodimeric complex has a smaller electrophoretic mobility than the homodimeric complexes such that both complexes can be resolved.^16^^,^^19^ To evaluate the partition of Max^∗WT^ and Max^∗Arg60Gln^ between their respective homo- and heterodimeric E-box-bound states, we integrated the intensities of both populations of complexes (Figure 5D). This revealed that the fraction of heterodimeric and specific complex is higher in the case of Max^∗Arg60Gln^. Collectively, our results demonstrate that the MAX p.Arg60Gln mutation leads to a b-HLH-LZ that will partition more readily as a heterodimeric and specific DNA complex with c-Myc than will its WT counterpart (Figure 6). Hence, it is likely that this mutation will increase the transcriptional activity of c-Myc in individuals bearing this mutation.

**Figure 7:**
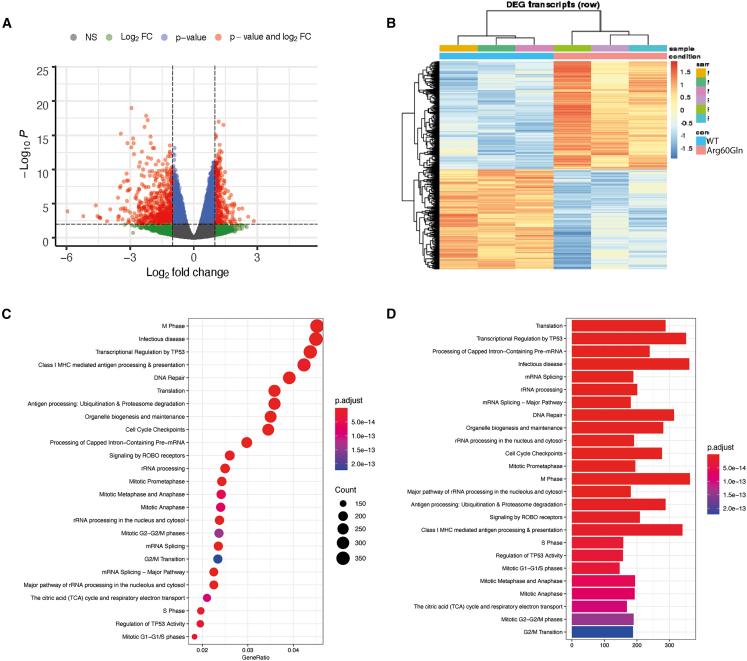
RNA sequencing shows dysregulated transcriptome in the presence of MAX^Arg60Gln^ (A) Volcano plot showing adjusted p value vs. log2 fold change of all expressed genes. Significantly up- or downregulated genes (padj < 0.01) are shown in blue except those that also have a log_2_fold change less than −1 or greater than +1, which are shown in red. (B) Heatmap based on the significantly differentially expressed transcripts. The samples (columns) and transcripts (rows) have been clustered to group things most alike between each biological replicate for MAX^WT^ and MAX^Arg60Gln^ transfected cells. (C) Dot plot showing the top 25 gene ontology (GO)-enriched terms based on GeneRatio. (D) Bar plot of the top 25 GO-enriched terms based on gene number. A recurrent *de novo* MAX p.Arg60Gln variant causes a syndromic overgrowth disorder through differential expression of c-Myc target genes.

### Transcriptome analysis reveals differential expression of c-Myc target genes

To better understand the transcriptional effect of the MAX^Arg60Gln^ variant on c-Myc target genes, we performed mRNA sequencing on HEK293 cells transfected with either MAX^WT^ or MAX^Arg60Gln^. Following alignment to the GRCh38 human genome with STAR, read counts were collected using Rsubread, and genes with less than 5 reads in all samples were discarded. This left 20,868 genes remaining for DGE analysis with DESeq2. Compared to WT transfected cells, we observed significant (padj < 0.05) upregulation of 3,122 genes, of which 250 had a log_2_FC > 1.0 (Table S1), and downregulation of 3,259 genes, of which 311 had a log_2_FC < −1.0, in MAX^Arg60Gln^ transfected cells (Figures 7A and 7B; Table S2). REACTOME pathway enrichment analysis of all differentially expressed genes identified those involved in translation (FDR 3.48 × 10^−16^) and metabolism of RNA (FDR 1.22 × 10^−15^) and proteins (FDR 1.00 × 10^−17^) were the most significantly enriched (Table S3). Enrichment analysis of up-regulated genes alone identified translation (FDR 1.62 × 10^−48^), metabolism of RNA (FDR 2.63 × 10^−40^) and RNA processing (FDR 4.82 × 10^−33^) to be most significantly enriched (Table S4), whereas RHO GTPase cycle (FDR 1.27 × 10^−21^), signaling by Rho GTPases, micro-GTPases, and RHOBTB3 (FDR 2.02 x 10^−20^), signaling by Rho GTPases (FDR 3.78 × 10^−20^), and signal transduction (FDR 1.68 × 10^−17^) were the most enriched pathways for down-regulated genes (Figures 7C and 7D; Table S5). A complete list of all significantly differentially expressed genes can be found in Table S6.

To determine if the genes identified were transcriptionally regulated by c-Myc, the list of DEGs were compared to a dataset of known Myc target genes identified by Kim et al.^24^ We found 1,175 of the 1,469 Myc direct target genes identified by Kim et al. were expressed in our HEK293 cell dataset, and of these, 565 (48.1%) were significantly differentially regulated by MAX^Arg60Gln^. We found 324/565 (57.3%) were up-regulated and 241/565 (42.7%) were down-regulated. Further interrogation of the significantly differentially expressed genes found that 83/324 (25.6%) up-regulated genes, including *CCND2*, had the CACGTG E-box promoter sequence, whereas 53/241 (22.0%) of the down-regulated genes had this sequence.

## Discussion

Through analysis of two large exome datasets, we have identified three individuals with a recurrent *de novo* heterozygous c.179G>A (p.Arg60Gln) variant in MAX with a macrocephaly- and polydactyly-associated syndrome. When ectopically expressed, the variant led to increased transcription and protein levels of CCND2 in HEK 293 cells. However, in contrast to previous studies linking CCND2 to macrocephaly-associated syndromes,^17^ this increase was not due to its stabilization but was due to increased transcription. Biophysical analysis of the Max^WT^ and Max^Arg60Gln^ b-HLH-LZ domains (Max^∗WT^ and Max^∗Arg60Gln^) confirmed a significant decrease in the affinity of the Max^∗Arg60Gln^ homodimer toward the canonical E-box sequence found at Myc target genes. Moreover, in the absence of DNA, we observed a more favorable formation of the Max^∗Arg60Gln^:c-Myc^∗^ b-HLH-LZ heterodimer compared to Max^∗WT^:c-Myc^∗^. In comparison to the WT, we also observed an increase in the potential of the Max^∗Arg60Gln^:c-Myc^∗^ heterodimer to bind to the canonical E-box sequence. Overall, the b-HLH-LZ of the Max^∗Arg60Gln^ variant was found to disfavor the formation of the repressive homodimeric E-box complex and synergistically favor the formation of the heterodimeric and activating DNA complex with c-Myc^∗^.

Max, as a homodimer, can compete for specific and non-specific transcriptional start sites (TSSs) with Myc (c-, L, N)/Max) and Mad (Mxd1, 2, 3, and 4)/Max heterodimers, as well as a plethora of other b-HLH and b-HLH-LZ with E-box sequences and non-specific DNA as target. Hence, the loss of affinity of the MAX^Arg60Gln^ variant for the E-box is predicted to allow for the binding of the aforementioned heterodimers. Moreover, the concomitant increase in free MAX^Arg60Gln^ vs. MAX^WT^ is not only expected to partition into a c-Myc/Max heterodimer but also as a Mad/Max and N-, L-Myc/Max heterodimers, depending on their respective and relative expression. According to the human protein Atlas (proteinatlas.org),^25^ c-Myc is expressed 10- to 20-fold higher than all members of the Mad family and Max, whereas N-Myc and L-Myc are barely detected. It is therefore reasonable to assume that the E-box-bound heterodimers with the MAX^Arg60Gln^ variant at promoters will include, in addition to c-Myc, a significant population of MDX/MAX^Arg60Gln^. Accordingly, RNA sequencing of HEK293 cells ectopically expressing the MAX^Arg60Gln^ variant revealed up- and downregulation in expression of c-Myc target genes vs. cells expressing MAX^WT^ at the same level.

To date, the association of macrocephaly with polydactyly has been observed in a range of disorders, including megalencephaly-polydactyly-polymicrogyria-hydrocephalus (MPPH; MIM: 615938), due to stabilizing mutations in *CCND2*,^17^ and megalencephaly-capillary malformation (MCAP; MIM: 602501) due to mutations of the PI3K-AKT-mTOR pathway upstream of CCND2.^26^ These so-called PIK3CA-related overgrowth syndromes (PROS), or mTORopathies result in hyperactivation of mTOR and stabilization of CCND2 through reduced GSK3β-mediated phosphorylation of CCND2-Thr280.^27^ In keeping with this, we observed increased CCND2 in cells transfected with MAX^Arg60Gln^, but this was not due to CCND2 stabilization but instead due to increased transcription. This finding was confirmed in individual P1 who showed increased levels of *CCND2* in peripheral blood RNA. While this is in contrast to mTOR pathway mutations, two studies have identified *de novo* gain-of-function variants in MYCN protogene, a bHLH transcription factor (*MYCN*) (MIM: 164840) associated with megalencephaly, ventriculomegaly, hypoplastic corpus callosum, intellectual disability, polydactyly, and neuroblastoma.^28^^,^^29^ The MYCN^Thr58Met^ and MYCN^Pro60Leu^ variants were found not to be phosphorylatable at Met58, leading to its accumulation in cells and appeared to induce *CCND1* and *CCND2* expression in neuronal progenitor and stem cells *in vitro*.

In comparison to Kato et al.,^28^ we observed no significant difference in *CCND1* expression in our RNA sequencing data, but significant upregulation (padj < 0.05) of *CCND2* (log2FC = 0.54) and *CCND3* (log2FC = 0.4*)*. As *CCND1* aberrations are known to contribute to neuroblastoma, this might explain why, as opposed to individuals with MYCN^Thr58Met^, individuals with the MAX^Arg60Gln^ variant do not have neuroblastoma, despite the overlapping phenotypes and underlying molecular mechanisms. These differences could be due to the different cell types analyzed; therefore, further research is required to better understand how these overlapping transcriptional networks are regulated, and in turn dysregulated, in disease.

The phenotypic spectrum of the three individuals, in addition to the macrocephaly and polydactyly, was broad, ranging from hypospadias, renal agenesis, single umbilical artery, flattened thoracic vertebrae, and death 1 h after birth in the most severely affected individual (P3) to perianal abscesses in P2. We hypothesize that these inconsistent phenotypes may be due to misregulation of additional genes downstream of c-Myc. This is supported by RNA sequencing analysis, which showed broad transcriptional dysregulation in the presence of the MAX^Arg60Gln^ variant. Similar broad transcriptional dysregulation has also been observed as a result of genomic aberration of transcription regulators that compete for the same E-box promoter sequence as Max and c-Myc. For example, a recent study showed loss of MITF, encoding the microphthalmia-inducing transcription factor, in a melanoma cell line led to 2,136 genes being differentially expressed, of which 1,516 showed at least a 2-fold change in expression.^30^ Loss-of-function mutations in microphthalmia-inducing transcription factor (MITF) cause a similar macrocephaly-associated syndrome known as COMMAD (coloboma, osteopetrosis, microphthalmia, macrocephaly, albinism, and deafness; MIM: 617306).^31^ Altogether, we hypothesize that in the presence of the MAX p.Arg60Gln variant, or biallelic loss-of-function mutations in *MITF*, c-Myc has reduced competition for the E-box sequence, resulting in dysregulated expression of downstream Myc-associated genes. This could go some way to explain the overlapping phenotypes observed between COMMAD and the cases described herein.

Currently, therapeutic options for individuals with this heterogeneous group of overgrowth syndromes are limited. mTOR inhibitors derived from rapamycin, namely everolimus and temsirolimus, have been used for the treatment of tuberous sclerosis complex-associated angiomyolipomas (MIM: 191100) and PTEN hamartoma tumor syndrome (PHTS; MIM: 601728) but with mixed results.^32^^,^^33^^,^^34^^,^^35^ Recent success has been achieved using alpelisib to treat PIK3CA-associated disorders, including CLOVE syndrome (MIM: 612918); however, this is only applicable to individuals harboring a *PIK3CA* mutation.^36^ While these therapeutics are unlikely to mediate the disease phenotypes resulting from mutations in *MAX*, therapies that modulate binding of c-Myc to the canonical E-box may have some promise. Of particular interest is a dominant-negative form of Myc, known as Omomyc-CPP, that has recently started clinical trials as a treatment for c-Myc-driven cancers.^37^ Given that the disease mechanism described in this study results in excessive c-Myc-driven transcription, it is possible that Omomyc could be an effective treatment for individuals with germline *MAX* mutations.

In summary, we have identified a recurrent germline *de novo* p.Arg60Gln variant in MAX as a cause of a macrocephaly- and polydactyly-associated syndrome. We provide evidence that the MAX p.Arg60Gln variant may cause the disease because of the loss or reduction of the repressing function of the MAX homodimer bound to the E-box sequence and to the concomitant increase in the binding of the c-Myc/Max heterodimer. This is proposed to result in less competition from MAX^Arg60Gln^ than from MAX^WT^ for the E-box promoter sequence and dysregulation of c-Myc-mediated transcription of target genes, including *CCND2*. Thus, unlike previously described macrocephaly-associated disorders, the mutation in *MAX* does not result in stabilized CCND2 but instead increases transcription of *CCND2*. Molecules that competitively bind the E-box sequence may therefore serve as an effective therapeutic for individuals harboring germline MAX-associated pathogenic variants.

## Data and code availability

All data are available from the corresponding authors on request. The p.Arg60Gln variant described in this manuscript has been submitted to ClinVar (accession number SCV002584947). The MAX^WT^ and MAX^Arg60Gln^ pDEST-510 plasmids used in this study have been deposited in Addgene with plasmid IDs #212944 and #212945, respectively. The fastq files generated from RNA sequencing have been deposited and are available from the NCBI Sequence Read Archive (SRA) under BioProject ID PRJNA1037166.

## Supplemental information

Data S1. Tables S1–S6

Document S2. Article plus supplemental information

## References

[1] Lüscher B., Vervoorts J. (2012). Regulation of gene transcription by the oncoprotein MYC. Gene.

[2] Blackwood E.M., Eisenman R.N. (1991). Max: a helix-loop-helix zipper protein that forms a sequence-specific DNA-binding complex with Myc. Science.

[3] Walz S., Lorenzin F., Morton J., Wiese K.E., von Eyss B., Herold S., Rycak L., Dumay-Odelot H., Karim S., Bartkuhn M. (2014). Activation and repression by oncogenic MYC shape tumour-specific gene expression profiles. Nature.

[4] Wolf E., Lin C.Y., Eilers M., Levens D.L. (2015). Taming of the beast: shaping Myc-dependent amplification. Trends Cell Biol..

[5] Montagne M., Beaudoin N., Fortin D., Lavoie C.L., Klinck R., Lavigne P. (2012). The Max b-HLH-LZ can transduce into cells and inhibit c-Myc transcriptional activities. PLoS One.

[6] Burnichon N., Cascón A., Schiavi F., Morales N.P., Comino-Méndez I., Abermil N., Inglada-Pérez L., de Cubas A.A., Amar L., Barontini M. (2012). MAX mutations cause hereditary and sporadic pheochromocytoma and paraganglioma. Clin. Cancer Res..

[7] Romero O.A., Torres-Diz M., Pros E., Savola S., Gomez A., Moran S., Saez C., Iwakawa R., Villanueva A., Montuenga L.M. (2014). MAX inactivation in small cell lung cancer disrupts MYC-SWI/SNF programs and is synthetic lethal with BRG1. Cancer Discov..

[8] Comino-Méndez I., Gracia-Aznárez F.J., Schiavi F., Landa I., Leandro-García L.J., Letón R., Honrado E., Ramos-Medina R., Caronia D., Pita G. (2011). Exome sequencing identifies MAX mutations as a cause of hereditary pheochromocytoma. Nat. Genet..

[9] Rapizzi E., Ercolino T., Canu L., Giaché V., Francalanci M., Pratesi C., Valeri A., Mannelli M. (2012). Mitochondrial function and content in pheochromocytoma/paraganglioma of succinate dehydrogenase mutation carriers. Endocr. Relat. Cancer.

[10] Deciphering Developmental Disorders Study (2015). Large-scale discovery of novel genetic causes of developmental disorders. Nature.

[11] Wright C.F., Campbell P., Eberhardt R.Y., Aitken S., Perrett D., Brent S., Danecek P., Gardner E.J., Chundru V.K., Lindsay S.J. (2023). Genomic Diagnosis of Rare Pediatric Disease in the United Kingdom and Ireland. N. Engl. J. Med..

[12] Vissers L.E.L.M., van Nimwegen K.J.M., Schieving J.H., Kamsteeg E.J., Kleefstra T., Yntema H.G., Pfundt R., van der Wilt G.J., Krabbenborg L., Brunner H.G. (2017). A clinical utility study of exome sequencing versus conventional genetic testing in pediatric neurology. Genet. Med..

[13] DeLano W.L. (2002).

[14] Sammak S., Hamdani N., Gorrec F., Allen M.D., Freund S.M.V., Bycroft M., Zinzalla G. (2019). Crystal Structures and Nuclear Magnetic Resonance Studies of the Apo Form of the c-MYC:MAX bHLHZip Complex Reveal a Helical Basic Region in the Absence of DNA. Biochemistry.

[15] Delattre P., Montagne M., Lavigne P. (2021). Methods of Expression, Purification, and Preparation of the c-Myc b-HLH-LZ for Its Biophysical Characterization. Methods Mol. Biol..

[16] Maltais L., Montagne M., Bédard M., Tremblay C., Soucek L., Lavigne P. (2017). Biophysical characterization of the b-HLH-LZ of DeltaMax, an alternatively spliced isoform of Max found in tumor cells: Towards the validation of a tumor suppressor role for the Max homodimers. PLoS One.

[17] Mirzaa G., Parry D.A., Fry A.E., Giamanco K.A., Schwartzentruber J., Vanstone M., Logan C.V., Roberts N., Johnson C.A., Singh S. (2014). De novo CCND2 mutations leading to stabilization of cyclin D2 cause megalencephaly-polymicrogyria-polydactyly-hydrocephalus syndrome. Nat. Genet..

[18] McDuff F.O., Naud J.F., Montagne M., Sauvé S., Lavigne P. (2009). The Max homodimeric b-HLH-LZ significantly interferes with the specific heterodimerization between the c-Myc and Max b-HLH-LZ in absence of DNA: a quantitative analysis. J. Mol. Recognit..

[19] Beaulieu M.E., McDuff F.O., Frappier V., Montagne M., Naud J.F., Lavigne P. (2012). New structural determinants for c-Myc specific heterodimerization with Max and development of a novel homodimeric c-Myc b-HLH-LZ. J. Mol. Recognit..

[20] Meier-Andrejszki L., Bjelić S., Naud J.F., Lavigne P., Jelesarov I. (2007). Thermodynamics of b-HLH-LZ protein binding to DNA: the energetic importance of protein-DNA contacts in site-specific E-box recognition by the complete gene product of the Max p21 transcription factor. Biochemistry.

[21] Brownlie P., Ceska T., Lamers M., Romier C., Stier G., Teo H., Suck D. (1997). The crystal structure of an intact human Max-DNA complex: new insights into mechanisms of transcriptional control. Structure.

[22] Ferré-D'Amaré A.R., Prendergast G.C., Ziff E.B., Burley S.K. (1993). Recognition by Max of its cognate DNA through a dimeric b/HLH/Z domain. Nature.

[23] Nair S.K., Burley S.K. (2003). X-ray structures of Myc-Max and Mad-Max recognizing DNA. Molecular bases of regulation by proto-oncogenic transcription factors. Cell.

[24] Kim J., Lee J.H., Iyer V.R. (2008). Global identification of Myc target genes reveals its direct role in mitochondrial biogenesis and its E-box usage in vivo. PLoS One.

[25] Uhlén M., Fagerberg L., Hallström B.M., Lindskog C., Oksvold P., Mardinoglu A., Sivertsson Å., Kampf C., Sjöstedt E., Asplund A. (2015). Proteomics. Tissue-based map of the human proteome. Science.

[26] Rivière J.B., Mirzaa G.M., O'Roak B.J., Beddaoui M., Alcantara D., Conway R.L., St-Onge J., Schwartzentruber J.A., Gripp K.W., Nikkel S.M. (2012). De novo germline and postzygotic mutations in AKT3, PIK3R2 and PIK3CA cause a spectrum of related megalencephaly syndromes. Nat. Genet..

[27] Kida A., Kakihana K., Kotani S., Kurosu T., Miura O. (2007). Glycogen synthase kinase-3beta and p38 phosphorylate cyclin D2 on Thr280 to trigger its ubiquitin/proteasome-dependent degradation in hematopoietic cells. Oncogene.

[28] Kato K., Miya F., Hamada N., Negishi Y., Narumi-Kishimoto Y., Ozawa H., Ito H., Hori I., Hattori A., Okamoto N. (2019). MYCN de novo gain-of-function mutation in a patient with a novel megalencephaly syndrome. J. Med. Genet..

[29] Nishio Y., Kato K., Mau-Them Frederic T., Futagawa H., Quélin C., Masuda S., Vitobello A., Otsuji S., Shawki H.H., Oishi H. (2023). Gain-of-function MYCN causes a megalencephaly-polydactyly syndrome manifesting mirror phenotypes of Feingold syndrome. HGG Adv..

[30] Dilshat R., Fock V., Kenny C., Gerritsen I., Lasseur R.M.J., Travnickova J., Eichhoff O.M., Cerny P., Möller K., Sigurbjörnsdóttir S. (2021). MITF reprograms the extracellular matrix and focal adhesion in melanoma. Elife.

[31] George A., Zand D.J., Hufnagel R.B., Sharma R., Sergeev Y.V., Legare J.M., Rice G.M., Scott Schwoerer J.A., Rius M., Tetri L. (2016). Biallelic Mutations in MITF Cause Coloboma, Osteopetrosis, Microphthalmia, Macrocephaly, Albinism, and Deafness. Am. J. Hum. Genet..

[32] Franz D.N., Lawson J.A., Yapici Z., Ikeda H., Polster T., Nabbout R., Curatolo P., de Vries P.J., Dlugos D.J., Voi M. (2018). Everolimus for treatment-refractory seizures in TSC: Extension of a randomized controlled trial. Neurol. Clin. Pract..

[33] Marsh D.J., Trahair T.N., Martin J.L., Chee W.Y., Walker J., Kirk E.P., Baxter R.C., Marshall G.M. (2008). Rapamycin treatment for a child with germline PTEN mutation. Nat. Clin. Pract. Oncol..

[34] Iacobas I., Burrows P.E., Adams D.M., Sutton V.R., Hollier L.H., Chintagumpala M.M. (2011). Oral rapamycin in the treatment of patients with hamartoma syndromes and PTEN mutation. Pediatr. Blood Cancer.

[35] Schmid G.L., Kässner F., Uhlig H.H., Körner A., Kratzsch J., Händel N., Zepp F.P., Kowalzik F., Laner A., Starke S. (2014). Sirolimus treatment of severe PTEN hamartoma tumor syndrome: case report and in vitro studies. Pediatr. Res..

[36] Venot Q., Blanc T., Rabia S.H., Berteloot L., Ladraa S., Duong J.P., Blanc E., Johnson S.C., Hoguin C., Boccara O. (2018). Targeted therapy in patients with PIK3CA-related overgrowth syndrome. Nature.

[37] Beaulieu M.E., Jauset T., Massó-Vallés D., Martínez-Martín S., Rahl P., Maltais L., Zacarias-Fluck M.F., Casacuberta-Serra S., Serrano Del Pozo E., Fiore C. (2019). Intrinsic cell-penetrating activity propels Omomyc from proof of concept to viable anti-MYC therapy. Sci. Transl. Med..

